# Microcirculatory dysfunction and dead-space ventilation in early ARDS: a hypothesis-generating observational study

**DOI:** 10.1186/s13613-020-00651-1

**Published:** 2020-03-24

**Authors:** Gustavo A. Ospina-Tascón, Diego F. Bautista, Humberto J. Madriñán, Juan D. Valencia, William F. Bermúdez, Edgardo Quiñones, Luis Eduardo Calderón-Tapia, Glenn Hernandez, Alejandro Bruhn, Daniel De Backer

**Affiliations:** 1 Department of Intensive Care, Fundación Valle del Lili - Universidad ICESI, Av. Simón Bolívar Cra. 98, Cali, Valle del Cauca Colombia; 2 Translational Medicine Laboratory for Critical Care and Advanced Trauma Surgery, Fundación Valle del Lili - Universidad Icesi, Cali, Colombia; 3 Departamento de Medicina Intensiva, Pontificia Universidad Católica de Chile, Santiago, Chile; 4 Department of Intensive Care, CHIREC Hospitals, Université Libre de Bruxelles, Brussels, Belgium

**Keywords:** Acute respiratory distress syndrome, Dead-space ventilation, *V*_D_/*V*_T_, Ventilation/perfusion mismatch, Microcirculation, Microcirculatory blood flow

## Abstract

**Background:**

Ventilation/perfusion inequalities impair gas exchange in acute respiratory distress syndrome (ARDS). Although increased dead-space ventilation (*V*_D_/*V*_T_) has been described in ARDS, its mechanism is not clearly understood. We sought to evaluate the relationships between dynamic variations in *V*_D_/*V*_T_ and extra-pulmonary microcirculatory blood flow detected at sublingual mucosa hypothesizing that an altered microcirculation, which is a generalized phenomenon during severe inflammatory conditions, could influence ventilation/perfusion mismatching manifested by increases in *V*_D_/*V*_T_ fraction during early stages of ARDS.

**Methods:**

Forty-two consecutive patients with early moderate and severe ARDS were included. PEEP was set targeting the best respiratory-system compliance after a PEEP-decremental recruitment maneuver. After 60 min of stabilization, hemodynamics and respiratory mechanics were recorded and blood gases collected. *V*_D_/*V*_T_ was calculated from the CO_2_ production ($V_{{{\text{CO}}_{2} }}$) and CO_2_ exhaled fraction ($F_{{{\text{ECO}}_{2} }}$) measurements by volumetric capnography. Sublingual microcirculatory images were simultaneously acquired using a sidestream dark-field device for an ulterior blinded semi-quantitative analysis. All measurements were repeated 24 h after.

**Results:**

Percentage of small vessels perfused (PPV) and microcirculatory flow index (MFI) were inverse and significantly related to *V*_D_/*V*_T_ at baseline (Spearman’s rho = − 0.76 and − 0.63, *p* < 0.001; *R*^2^ = 0.63, and 0.48, *p* < 0.001, respectively) and 24 h after (Spearman’s rho = − 0.71, and − 0.65; *p* < 0.001; *R*^2^ = 0.66 and 0.60, *p* < 0.001, respectively). Other respiratory, macro-hemodynamic and oxygenation parameters did not correlate with *V*_D_/*V*_T_. Variations in PPV between baseline and 24 h were inverse and significantly related to simultaneous changes in *V*_D_/*V*_T_ (Spearman’s rho = − 0.66, *p* < 0.001; *R*^2^ = 0.67, *p* < 0.001).

**Conclusion:**

Increased heterogeneity of microcirculatory blood flow evaluated at sublingual mucosa seems to be related to increases in *V*_D_/*V*_T_, while respiratory mechanics and oxygenation parameters do not. Whether there is a cause–effect relationship between microcirculatory dysfunction and dead-space ventilation in ARDS should be addressed in future research.

## Background

Acute respiratory distress syndrome (ARDS) is a form of acute respiratory failure characterized by pulmonary inflammation leading to increased capillary and epithelial permeability, with subsequent loss of aerated lung tissue and increased lung stiffness [1]. These alterations lead to imbalances between ventilation and perfusion relationships, which finally result in hypoxemia and impaired carbon dioxide clearance.

An optimal ventilation-to-perfusion (*V*_A_/*Q*) ratio (0.1 < *V*_A_/*Q* < 10) is necessary to ensure a normal gas exchange [2–4]. Typically, it has been considered that pulmonary perfusion in ARDS occurs in non-ventilated (*V*_A_/*Q* < 0.005) or poorly ventilated (0.005 < *V*_A_/*Q* < 0.1) lung units, which, in turn, results in vasoconstriction of perfusing arterioles [5]. Such *V*_A_/*Q* mismatch in some lung regions in which perfusion largely exceeds ventilation, account for hypoxemia, which is the clinical hallmark of ARDS [1, 6, 7]. Nevertheless, distribution of ventilation to poorly perfused (10 < *V*_A_/*Q* < 100), severely hypoperfused (*V*_A_/*Q* > 100) or non-perfused (*V*_A_/*Q* ~ ∞) lung units might also occur in patients with ARDS [3] and indeed, increases in *V*_D_/*V*_T_ have been strongly related with adverse clinical outcomes [8–10]. Importantly, high *V*_A_/*Q* and *V*_A_/*Q* ~ ∞ ratios corresponding to lung regions where ventilation largely exceeds perfusion, account for carbon dioxide retention [5]. Increases in high *V*_A_/*Q* and/or dead-space lung units have been classically attributed to alveolar overdistention with the subsequent compression of intra-alveolar vessels in the non-dependent lung areas [8, 11]. Nevertheless, increased dead-space ventilation has also been described in patients subjected to protective ventilation strategies with low plateau pressures [9, 10], which suggest that mechanisms different to alveolar overdistention should be implied.

In normal conditions, the heterogeneity of systemic microcirculatory blood flow distribution is negligible [12]. Nevertheless, severe inflammation can induce microcirculatory alterations [13, 14] determining alterations in oxygen extraction capabilities by the tissues and contributing to the development of multiple organ dysfunction [14]. Although there are many technical limitations to directly evaluate pulmonary microcirculation [15], heterogeneity of microvascular blood flow at pulmonary level could contribute to imbalances between ventilation and perfusion relationships. Thus, considering microcirculatory dysfunction during inflammatory conditions as a generalized phenomenon, which may involve systemic and pulmonary vascular beds, we hypothesized that alterations in microvascular blood flow distribution evaluated at the sublingual mucosa as representative of an extra-pulmonary territory could be related to variations in dead-space ventilation *V*_D_/*V*_T_ during early phases of moderate and severe ARDS.

## Methods

This prospective observational study was conducted in a 90-bed mixed ICU from a university hospital. The local Ethical and Biomedical Research Committee approved the study (Fundación Valle del Lili EBRC protocol number: 0628; approval number: 038-7, 2013). A written informed consent was waived as no invasive procedures or new interventions were used. We daily screened all patients under mechanical ventilation in the ICU during a 24-month period, searching for those with moderate and severe ARDS. To avoid the selection of cases with transitory hypoxemia simulating ARDS, patients were enrolled only after successfully completing a two-step selection process [16, 17]: (a) first, patients mechanically ventilated through an endotracheal tube with a PEEP ≥ 5 and FiO_2_ ≥ 0.5 for at least 12 h and meeting the moderate and severe ARDS criteria according to Berlin Consensus definitions [1] were declared potentially eligible; (b) then, potential candidates were subjected to a FiO_2_ trial at 1.0 while maintaining PEEP ≥ 10 (to sustain a SpO_2_ ≥ 88%, but ensuring peak inspiratory and plateau pressures < 35 and 28 cmH_2_O, respectively) for at least 30 min, after which, new arterial blood gases were collected. Those patients maintaining a PaO_2_/FiO_2_ ≤ 200 after such PEEP/FiO_2_ trial and with < 48 h of evolution of ARDS were finally included. The exclusion criteria were: < 18 years of age, pregnancy state, history of neuromuscular diseases, moderate and severe COPD (defined as FEV_1_ < 80% predicted); history of intubation due to COPD exacerbation, receiving domiciliary oxygen or long-term use of steroids because COPD; history of congestive heart failure or any acute ischemic cardiac condition. A patient was also excluded when limitation of therapeutic effort orders were given.

### Study protocol

After fulfilling the two-step selection process, patients selected were connected to a mainstream CO_2_ sensor and this in turn to a volumetric capnography module (Infinity EtCO_2_ + respiratory mechanics module, Dräger Medical Systems, Telford, USA). Mechanical ventilation parameters were adjusted after a stepwise alveolar recruitment maneuver, as it will be detailed later. After a 60 min of stabilization period, we started capnography measurements while sublingual microcirculatory images were simultaneously acquired, such as detailed thereafter. A new set of measurements was obtained 24 h after. Arterial and mixed venous blood samples (when available) were drawn for gases analysis (ABL300, Radiometer; Copenhagen, Denmark) at T0 and 24 h after (T24). In all the cases, the attending physicians decided on the type of hemodynamic monitoring to use. Complete respiratory and hemodynamic parameters were also registered simultaneously.

### Recruitment maneuvers, PEEP adjustment and mechanical ventilation settings

At the time in which this study was performed, the local protocol included an initial recruitment maneuver to adjust PEEP in patients with severe ARDS. Thus, patients were subjected to a stepwise recruitment maneuver with progressive PEEP increases until a peak pressure of 50 cmH_2_O while maintaining a driving pressure of 15 cmH_2_O, as described elsewhere [18, 19]. Once obtained the maximal peak pressure, it was sustained during 2 min whereupon a decremental PEEP titration trial was conducted in steps of 2 cmH_2_O at 2 min interval from 22 to 8 cmH_2_O registering the corresponding compliance of the respiratory system (*C*_RS_). After such a PEEP titration, a new alveolar recruitment was performed until a peak pressure of 50 cmH_2_O while maintaining a driving pressure of 15 cmH_2_O during 1 min, to finally adjust the definitive ventilatory settings. Definitive PEEP was set at the corresponding best *C*_RS_ plus 2 cmH_2_O. If falls in C_RS_ were observed in two consecutive downsteps, then the PEEP level was set at the highest compliance plus 2 cmH_2_O. According to the local protocol, the recruitment maneuver was stopped if one or more of following signs were observed: heart rate > 150 or < 60 bpm; decrease of mean arterial pressure < 65 mmHg or systolic pressure < 90 mmHg; acute atrial fibrillation, atrial flutter or ventricular tachycardia.

Thereafter, mechanical ventilation was set in volume-controlled mode or in pressure-controlled, according to the selection of the attending physician. In the first case, ventilation was set at Vt of 6 ml/kg of predicted body weight maintaining plateau pressures < 28 cmH_2_O, flow of 60 l/min, inspiratory pause of 0.5 s, *I*:*E* ratio of 1:1 to 1:2, respiratory rate to match the minute ventilation previous to the recruitment maneuver, FiO_2_ necessary for SpO_2_ ≥ 90 and ≤ 95% and PEEP adjusted as indicated above. If plateau pressures were > 28 cmH_2_O, then Vt was reduced to a minimum of 4 ml/kg of predicted body weight. For those ventilated in pressure-controlled mode, driving pressure was adjusted to maintain Vt 6 ml/kg of predicted body weight (or less if Vt/*C*_RS_ > 18), *I*:*E* ratio 1:1 to 1:2, minute ventilation matching that previous to the recruitment maneuver, peak inspiratory pressure ≤ 35 cmH_2_O, and FiO_2_ and PEEP adjusted as indicated above.

### Pulmonary dead-space fraction measurements

After an automatic purge and calibration procedure, a mainstream CO_2_ sensor was placed between the ventilator circuit and the patient connection. This sensor was in turn connected to a volumetric capnography module (Infinity EtCO_2_ + respiratory mechanics module, Dräger Medical Systems, Telford, USA). After selection of the ventilator settings, a 60 min of stabilization period was allowed before to start the measurements. Data trend for CO_2_ production ($V_{{{\text{CO}}_{2} }}$) and exhaled minute ventilation (*V*_E_) were averaged over 5 min. $V_{{{\text{CO}}_{2} }}$ measurements were obtained at standard temperature and pressure, and dry (STPD), whereby a correction factor of 0.863 mmHg l/ml was used to convert to body temperature, and pressure, saturated (BTPS). The fraction of exhaled CO_2_ ($F_{{{\text{ECO}}_{2} }}$) was calculated dividing the $V_{{{\text{CO}}_{2} }}$ by the *V*_E_ (Eq. 1):

1 $$F_{{{\text{ECO}}_{2} }} = \frac{{V_{{{\text{CO}}_{2} }} }}{{V_{{{\text{E}} }} }} .$$

Exhaled CO_2_ pressure ($P_{{{\text{ECO}}_{2} }}$) was then calculated as the product between the $F_{{{\text{ECO}}_{2} }}$ and the barometric pressure minus the water vapor pressure (Eq. 2):

2 $$P_{{{\text{ECO}}_{2} }} = F_{{{\text{ECO}}_{2} }} . \left( {P_{{{\text{B}} }} - 47} \right),$$

where *P*_B_ corresponds to the local barometric pressure (i.e., 682 mmHg). Subsequently, *V*_D_/*V*_T_ was calculated by the Enghoff modification of the Bohr equation (Eq. 3):

3 $$\frac{{V_{\text{D}} }}{{V_{\text{T}} }} = (P_{{{\text{aCO}}_{2} }} - P_{{{\text{ECO}}_{2} }} ) / P_{{{\text{aCO}}_{2} }} .$$

All measurements of $V_{{{\text{CO}}_{2} }}$ performed by the module were automatically corrected for circuit compression, as described elsewhere [20].

### Sublingual microcirculation assessment

A sidestream dark-field (SDF) imaging device (Micro Scan; MicroVision Medical, Amsterdam, the Netherlands) was used to explore the sublingual microcirculation simultaneously to dead-space fraction measurements, ventilatory mechanics and oxygenation parameters at both inclusion and 24 h after. A cutoff value of 20 μm was used to classify vessels as large or small. Continuous flows were considered as normal while intermittent and stopped flows were considered as abnormal. According to the consensus for the evaluation of microcirculation, we calculated the proportion of small vessels perfused (PPV), the total vascular density (TCD) and the functional capillary density (FCD) [21]. A heterogeneity index of microcirculatory blood flow was also calculated as the difference between maximal and minimal PPV values in five different mucosa areas divided by its own mean value (see Additional file 1). Additionally, we reported the microvascular flow index (MFI). A detailed description about microcirculatory blood flow assessment is provided in Additional file 1.

### Statistical analysis

Sample size calculation is described in Additional file 1. Distribution of data was tested using the Kolmogorov–Smirnov test. Non-parametric test for related samples were used to evaluate the differences on hemodynamic, respiratory, capnometry and microcirculatory blood flow parameters between baseline and 24 h after. The relationships between the *V*_D_/*V*_T_, percentage of small vessels perfused (PPV), and microcirculatory blood flow index (MFI) were evaluated by the Spearman rho test. Other bivariate correlations between *V*_D_/*V*_T_, PaO_2_/FiO_2_, and respiratory mechanics were also performed using Spearman rho test. Additionally, simple linear regression models with linear and quadratic terms and their respective coefficients of determination (*R*^2^) were used to evaluate the relationship between each microcirculatory, respiratory mechanics or oxygenation parameter and the *V*_D_/*V*_T_ at both baseline and 24 h after.

Finally, we calculated the delta of variation of *V*_D_/*V*_T_ and PPV measurements between baseline and 24 h after. Then, a Spearman rho was used to evaluate the correlation between *V*_D_/*V*_T_ and PPV dynamic variations from baseline to day-1. Furthermore, a simple linear regression model with quadratic term and its respective coefficient of determination (*R*^2^) was used to evaluate the relationship between variations in PPV and *V*_D_/*V*_T_ from baseline to 24 h after. Data are presented as median [percentiles 25–75]. A *p* value ≤ 0.05 (2-tailed) was considered significant.

## Results

**Fig. 1:**
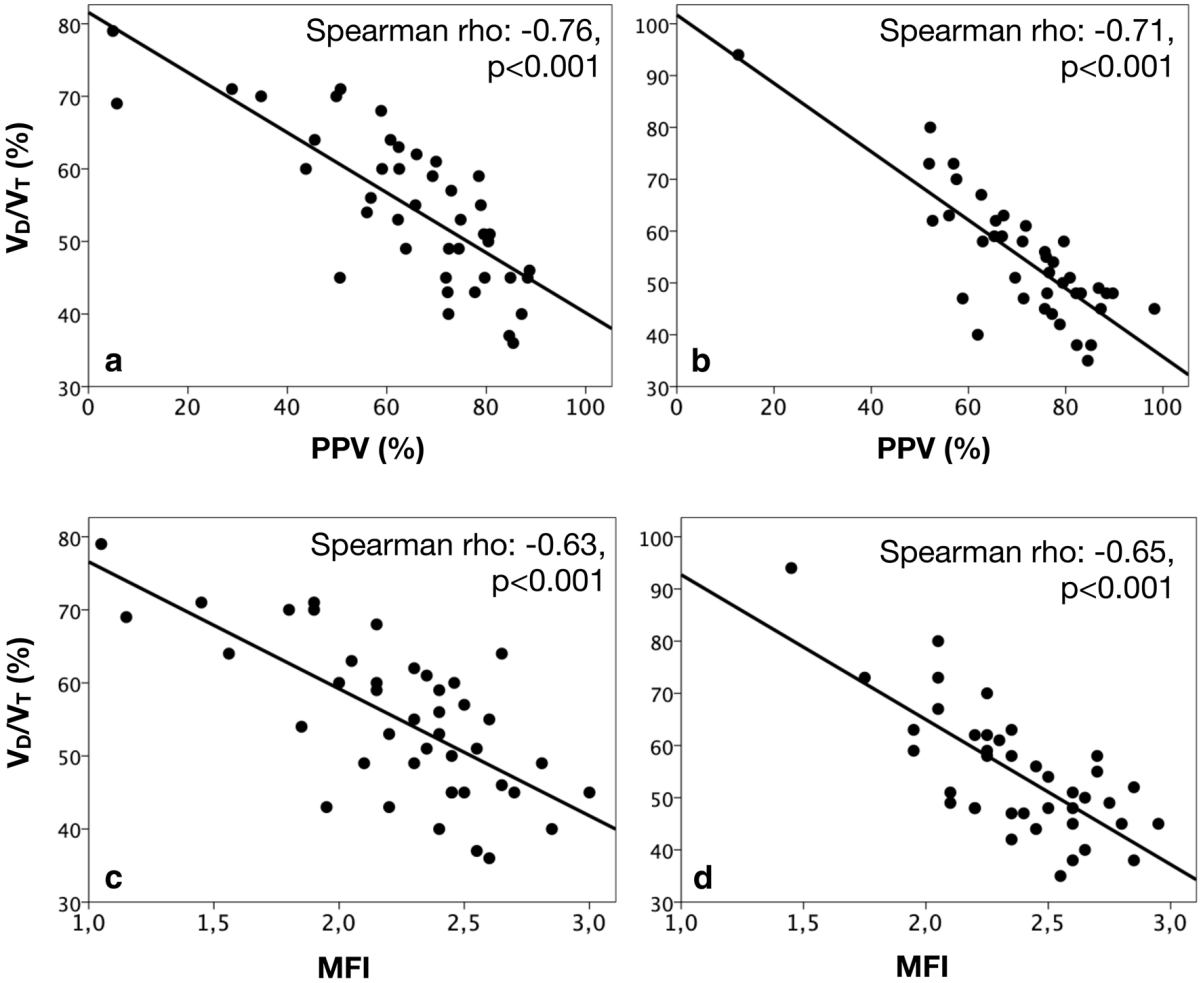
Relationships between pulmonary dead-space fraction (*V*_D_/*V*_T_) and the microcirculatory blood flow at baseline and 24 h after. **a** Scatter plot depicting the correlation between pulmonary dead-space fraction (*V*_D_/*V*_T_) and the proportion of small vessels perfused at baseline. **b** Scatter plot depicting the correlation between pulmonary dead-space fraction (*V*_D_/*V*_T_) and the proportion of small vessels perfused 24 h after. **c** Scatter plot depicting the correlation between pulmonary dead-space fraction (*V*_D_/*V*_T_) and MFI at baseline. **d** Scatter plot depicting the correlation between pulmonary dead-space fraction (*V*_D_/*V*_T_) and MFI 24 h after. PPV: percentage of small vessels perfused; *V*_D_*/V*_T_: pulmonary dead-space fraction; HI: heterogeneity index of microcirculatory blood flow; MFI: microcirculatory blood flow index

**Fig. 2:**
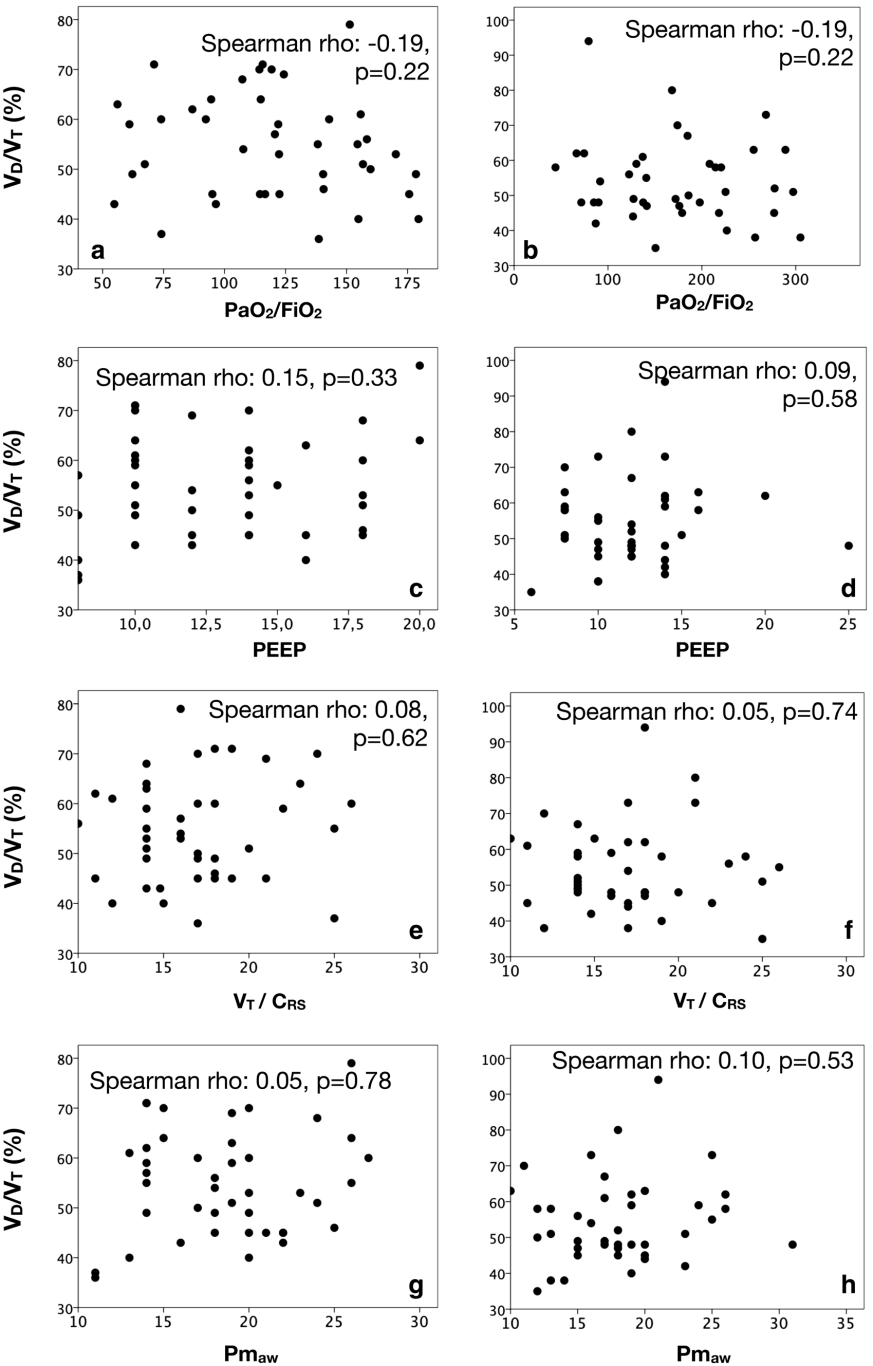
Relationships between pulmonary dead-space fraction (*V*_D_/*V*_T_) and some respiratory mechanics and oxygen parameters at baseline and 24 h after. **a** Scatter plot depicting the correlation between pulmonary dead-space fraction (*V*_D_/*V*_T_) and the PaO_2_/FiO_2_ ratio at baseline. **b** Scatter plot depicting the correlation between pulmonary dead-space fraction (*V*_D_/*V*_T_) and the PaO_2_/FiO_2_ ratio 24 h after. **c** Scatter plot depicting the correlation between pulmonary dead-space fraction (*V*_D_/*V*_T_) and PEEP levels at baseline. **d** Scatter plot depicting the correlation between pulmonary dead-space fraction (*V*_D_/*V*_T_) and PEEP levels 24 h after. **e** Scatter plot depicting the correlation between pulmonary dead-space fraction (*V*_D_/*V*_T_) and the *V*_T_/*C*_RS_ at baseline. **f** Scatter plot depicting the correlation between pulmonary dead-space fraction (*V*_D_/*V*_T_) and the *V*_T_/*C*_RS_ 24 h after. **g** Scatter plot depicting the correlation between pulmonary dead-space fraction (*V*_D_/*V*_T_) and Pm_aw_ at baseline. **h** Scatter plot depicting the correlation between pulmonary dead-space fraction (*V*_D_/*V*_T_) and Pm_aw_ 24 h after. PaO_2_/FiO_2_ ratio: arterial oxygen partial pressure to oxygen inspiratory fraction; PEEP: positive end-expiratory pressure; *V*_T_/*C*_RS_: tidal volume-to-respiratory system compliance ratio (i.e., driving pressure); Pm_aw_: mean pressure of the airway

A total of 42 patients with moderate and severe ARDS were included in the study. A complete flowchart detailing the selection process is shown in Additional file 1: Figure S1, while a STROBE statement checklist for observational studies is provided in Additional file 1: Table S1. Mortality at day-28 and day-90 were 31% and 45.2%, respectively. The ICU length of stay was 18.0 [11.8–26.3] days. General characteristics are presented in Table 1, while hemodynamics, respiratory mechanics, blood gases analysis, pulmonary dead-space fraction and microcirculatory blood flow parameters at baseline and 24 h after are presented in Table 2. We observed an inverse and significant relationship between PPV and *V*_D_/*V*_T_ at both baseline (Spearman rho = − 0.76, *p* < 0.001; *R*^2^ = 0.63, *p* < 0.001) and 24 h after (Spearman rho = − 0.71, *p* < 0.001; *R*^2^ = 0.66, *p* < 0.001) (Fig. 1a, b). Similar findings were observed between *V*_D_/*V*_T_ and the microcirculatory flow index at baseline (Spearman rho = − 0.63, *p* < 0.001; *R*^2^ = 0.48, *p* < 0.001) and 24 h after (Spearman rho = − 0.65, *p* < 0.001; *R*^2^ = 0.60, *p* < 0.001) (Fig. 1c, d). There were no significant correlations between *V*_D_/*V*_T_ and other respiratory mechanics and oxygenation parameters (Fig. 2, Additional file 1: Table S2).

**Table 1:** General characteristics

	All patients
Age	48 (33–63)
Sex, male (%)	24 (57.1)
APACHE II	21.5 (17.0 27.3)
SOFA, day 1	11.0 (7.8–13.3)
Coexisting conditions	
Hypertension	8 (19.0)
Diabetes	5 (11.9)
Hepatic disease	5 (13.2)
Chronic renal failure	3 (7.3)
Cancer	6 (14.3)
Immunosuppression	6 (14.3)
BMI (kg/m^2^)	20.7 (20.3–22.7)
Risk factors for ARDS	
Sepsis	28 (66.7)
Trauma	5 (13.2)
Pneumonia	13 (34.2)
Gastric aspiration	6 (15.8)
Other	11 (28.9)
Vasopressors, *n* (%)	26 (61.9)
Steroids, *n* (%)	15 (35.7)
TRR, *n* (%)	8 (19.5)
Prone position, *n* (%)	10 (23.8)
Muscular paralysis	34 (80.9)
Time since ICU admission to ARDS onset, hours	96 (24–168)
Cumulated resuscitation fluids, ml	2.300 (1.075–4.735)
Total cumulative fluids at ARDS diagnosis, ml	6.361 (2.182–13.023)
Mortality	
At day 28	13 (31.0)
At day 90	19 (45.2)

**Table 2:** Hemodynamics, respiratory mechanics, blood gases analysis, $V_{{{\text{CO}}_{2} }}$, *V*_D_/*V*_T_ and microcirculatory parameters at baseline and 24 h after

	Baseline	24 h	*p*
Hemodynamics			
HR, bpm	104 (94–121)	100 (89–115)	0.43
MAP, mmHg	74 (69–87)	75 (67–92)	0.35
CVP, mmHg, n	12 (8–14)	12 (7–14)	0.96
PAOP, mmHg, n	14 (12–16), 22	19 (16–22), 24	0.03
PAPm, mmHg, n	36 (42–46), 22	30 (26–41), 24	0.13
PVR, dyn.s/cm^5^, n	298 (250–440), 22	299 (211–360), 24	0.86
Cardiac index, (l/min/m^2^), *n*	4.2 (3.4–4.5), 22	3.9 (3.5–4.4), 24	0.30
Norepinephrine, (ugr/kg/min), *n*	0.10 (0.05–0.27), 18	0.05 (0.03–0.34), 18	0.09
Respiratory mechanics and blood gases			
*V*_T_, ml	369 (325–415)	363 (312–404)	0.56
*V*_T_, ml/kg	6.5 (6.1–6.9)	6.6 (6.4–6.9)	0.56
RR	24 (20–26)	25 (22–27)	0.17
PEEP	12 (10–15)	12 (10–14)	0.07
*P*_Peak_	30 (27–33)	29 (26–34)	0.57
Pm_aw_	19 (14–21)	18 (15–20)	0.36
*V*_T_/*C*_RS_	17 (14–19)	16 (14–18)	0.42
pH	7.28 (7.21–7.32)	7.35 (7.26–7.39)	< 0.001
PaO_2_, mmHg	79.1 (69.7–95.5)	86.6 (72.6–109.2)	0.06
PaCO_2_, mmHg	46.6 (39.9–56.3)	42.3 (34.6–47.6)	0.003
HCO_3_, mmol/L	20.9 (19.1–24.2)	20.7 (17.8–26.5)	0.78
BE, mmol/L	− 6.4 (− 8.8 to − 2.3)	− 5.9 (− 8.4 to − 0.05)	0.03
PaO_2_/FiO_2_	119 (93–148)	173 (124–222)	< 0.001
PvO_2_, mmHg	48.7 (42.3–54.1)	40.8 (36.9–47.6)	0.004
PvCO_2_, mmHg	48.2 (41.6–59.3)	44.6 (36.1–53.6)	0.003
ScvO_2_, %	78.0 (70.9–80.9)	75.2 (62.3–81.7)	0.17
Capnography/expired CO_2_			
$P_{{{\text{ECO}}_{2} }}$, mmHg (BTPS)	21.2 (18.4–24.6)	19.3 (14.8–21.6)	0.01
$F_{{{\text{ECO}}_{2} }}$	0.034 (0.029–0.039)	0.030 (0.023–0.034)	0.01
$V_{{{\text{CO}}_{2} }}$, ml/min (STPD)	320 (250–405)	259 (201–380)	0.28
*V*_TE_, ml	372 (325–415)	302 (240–440)	0.39
*V*_D_/*V*_T_	54 (45–61)	51 (47–61)	0.44
Microcirculatory blood flow parameters			
PPV, %	69.6 (57.8–79.2)	75.7 (62.8–81.5)	0.01
LVP, %	90.9 (82.3–96.5)	90.3 (85.3–96.8)	0.52
FCD, *n* vessels/mm^2^	6.6 (5.1–7.5)	6.9 (5.5–8.2)	0.03
TCD, *n* vessels/mm^2^	11.6 (10.8–12.6)	12.3 (11.0–13.3)	0.08
MFI	2.35 (2.08–2.50)	2.35 (2.20–2.60)	0.06
Heterogeneity index	0.38 (0.27–0.65)	0.28 (0.19–0.45)	0.08

HR: heart rate; MAP: mean arterial pressure; CVP: central venous pressure; PAOP: pulmonary artery occlusion pressure; PAPm: mean pulmonary artery pressure; PVR: pulmonary vascular resistance; *V*_T_: tidal volume; PEEP: positive end-expiratory pressure; *P*_Peak_: peak respiratory pressure; Pm_aw_: mean pressure of the airway; *V*_T_/*C*_RS_: tidal volume to respiratory system compliance (driving pressure); PaO_2_: arterial oxygen partial pressure; PaCO_2_: arterial carbon dioxide partial pressure; HCO_3_: bicarbonate; BE: base excess; PaO_2_/FiO_2_ ratio: arterial oxygen partial pressure to oxygen inspiratory fraction; PvO_2_: venous oxygen partial pressure; PvCO_2_: venous carbon dioxide partial pressure; SvO_2_: venous hemoglobin oxygen saturation; $P_{{{\text{ECO}}_{2} }}$: exhaled carbon dioxide pressure; BTPS: body temperature, and pressure, saturated; STPD: standard temperature and pressure, dry; $F_{{{\text{ECO}}_{2} }}$: exhaled carbon dioxide fraction; *V*_TE_: exhaled tidal volume; *V*_D_/*V*_T_: pulmonary dead-space fraction; PPV: percentage of small vessels perfused; LVP: percentage of large vessels perfused; FCD: functional capillary density; TCD: total capillary density; MFI: microvascular blood flow index

**Fig. 3:**
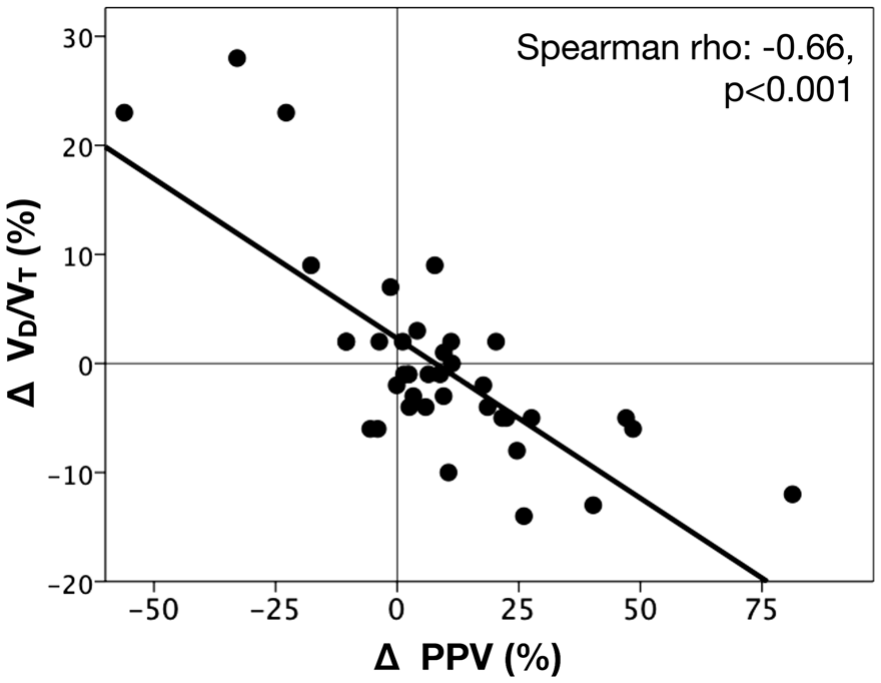
Relationships between dynamic variations in pulmonary dead-space fraction (*V*_D_/*V*_T_) and microcirculatory blood flow. Scatter plot depicting the correlation between variations in pulmonary dead-space fraction (Δ-*V*_D_/*V*_T_) vs. percentage of change in small vessels perfused (Δ-PPV) between baseline and 24 h after

A significant relationship was observed between the variation in *V*_D_/*V*_T_ and the percentage of variation of PPV from baseline measurements to 24 h after (Spearman rho = − 0.66, *p* < 0.001; *R*^2^ = 0.67, *p* < 0.001) (Fig. 3, Additional file 1: Figure S2). Additional information about survivors and non-survivors at day-90 is provided in Additional file 1: Table S3.

## Discussion

After simultaneous calculation of dead-space fraction by volumetric capnography and exploration of sublingual microcirculation by the SDF technique during the early stages of moderate and severe ARDS, we retrieved two hypothesis-generating observations: (a) *V*_D_/*V*_T_ is inverse and significantly related with sublingual microcirculatory blood flow distribution, while PEEP levels, respiratory airway pressures, PaO_2_/FiO_2_ and lung strain surrogates (*V*_T_/*C*_RS_) do not; (b) *V*_D_/*V*_T_ variations were closely related with dynamic changes in the microcirculatory blood flow distribution observed at sublingual mucosa.

Other mechanisms unrelated to shunt-induced hypoxemia could be implicated in gas exchange abnormalities and in the onset of pulmonary and extra-pulmonary multiorgan dysfunction in ARDS. Distribution of ventilation to poorly perfused (10 < *V*_A_/*Q* < 100), severely hypoperfused (*V*_A_/*Q* > 100) or non-perfused, i.e., true dead-space ventilation (*V*_A_/*Q* ~ ∞) lung units, can also contribute to gas exchange disturbances and it might be a key piece in the pathophysiology of ARDS. Some studies in the past demonstrated the occurrence of increased pulmonary dead-space fraction in patients with acute hypoxemic respiratory failure [3, 22, 23], and highlighted the apparent relationship between high *V*_D_/*V*_T_ and increased mortality [8]. Nevertheless, early studies included patients under non-lung-protective ventilation strategies, in which over-inflation leading to capillary collapse could explain *V*_A_/*Q* mismatching with the resultant increased *V*_D_/*V*_T_ [24]. Remarkably, later studies in ARDS patients subjected to lung-protective ventilation, also demonstrated the occurrence of increases in *V*_D_/*V*_T_ and confirmed its consistent relationship with worse clinical outcomes [9, 10, 25]. In agreement with this, we observed increases in *V*_D_/*V*_T_ at baseline and 24 h after in patients with moderate and severe ARDS. Importantly, we did not find any relationship between *V*_D_/*V*_T_ and variables suggesting vascular collapse related to alveolar overdistention or increased pulmonary strain (e.g., *V*_T_/*C*_RS_), although admittedly, controlling airway pressures and *V*_T_/*C*_RS_ (driving pressure) could not prevent alveolar overdistention because the inhomogeneous lung compromise in ARDS [26].

Relationships between microvascular blood flow and *V*_D_/*V*_T_ have not been widely studied because of technical limitations to directly evaluate pulmonary microcirculation [15]. Our data suggest an apparent relationship between microcirculatory dysfunction and dead-space ventilation. Admittedly, sublingual mucosa and pulmonary circulation are two dissimilar vascular beds with particular regulating mechanisms. Nevertheless, during inflammatory conditions, microcirculatory dysfunction is a generalized phenomenon involving simultaneously most vascular beds [14], although with different effects depending on the territory studied [27]. Microcirculatory alterations have been described in autopsies and biopsies from lungs of patients with acute hypoxemic respiratory failure [28, 29] and angiograms performed through pulmonary artery catheters demonstrated filling defects attributable to macro- and micro-emboli [30, 31]. Increases in *V*_D_/*V*_T_ in our patients were well correlated with alterations in microcirculatory blood flow distribution detected in a non-pulmonary vascular bed. Such observation could pose the hypothesis about heterogeneity of microvascular blood flow contributing to inequalities in *V*_A_/*Q* relationships. Indeed, variations of *V*_D_/*V*_T_ from baseline to 24 h after were closely related with dynamic changes in microcirculatory blood flow distribution at sublingual mucosa, which reinforce the strength of such relation. Nevertheless, whether pulmonary microvascular alterations or other organ-specific microvascular blood flow can be evaluated or estimated through evaluation of an extra-pulmonary microvascular bed can result highly controversial [15].

In normal conditions, heterogeneity of microvascular blood flow is negligible [12] and matching of perfusion to metabolism usually improves during hypoxic or low-flow states [32]. However, during inflammatory conditions, heterogeneity of microcirculation increases as consequence of the interruption of blood flow of individual capillaries causing derangements in the oxygen extraction capabilities, thus contributing to organ failure. In agreement with this, we observed important microcirculatory alterations consisting in decreased PPV, reduced FCD and increased heterogeneity of blood flow, which were in turn linked to more severe extra-pulmonary organ dysfunction quantified by SOFA score (see Additional file 1: Table S3).

Pathophysiological mechanisms increasing *V*_D_/*V*_T_ in ARDS are quite complex. An increased *V*_D_/*V*_T_ reflects a global assessment of abnormal gas exchange, but not simply the contribution of discrete high *V*_A_/*Q* regions and true anatomic dead space (*V*_A_/*Q* ~ ∞). Although the patchy pattern of vascular damage is a phenomenon clearly recognized in ARDS [26], no studies demonstrated that damaged areas necessarily receive substantial ventilation, as would be necessary to explain regions of high *V*_A_/*Q* ratio. Using the multiple inert gas elimination technique (MIGET) to evaluate the fractional contribution of each *V*_A_/*Q* abnormality (shunt, mid-range *V*_A_/*Q* heterogeneity, high *V*_A_/*Q*, and anatomic dead space) on total *V*_D_/*V*_T_ at progressively high PEEP levels, Coffey et al. [33] demonstrated similar *V*_D_/*V*_T_ values at different PEEP levels mediated by very different physiologic abnormalities, although certainly, higher PEEP values were consistently related to high *V*_A_/*Q* peaks. Our results might add more complexity to the pathophysiology on increased *V*_D_/*V*_T_ in ARDS suggesting the contribution of altered microcirculatory blood flow distribution on the increase of high *V*_A_/*Q* units.

Routine assessment of pulmonary gas exchange in ARDS is based on analysis of oxygen and carbon dioxide partial pressures. These variables, although sensitive to intrapulmonary factors (e.g., shunt and *V*_A_/*Q* matching), could be also altered by extra-pulmonary elements such as cardiac output, oxygen consumption, minute ventilation and inspired oxygen fraction.

*V*_D_/*V*_T_ values can widely vary according to the method used to estimate it [34]. Previous studies used the Enghoff modification of the Bohr equation (VD_Enghoff_) in patients with ARDS [8–10, 25]. Nevertheless, this method could overestimate the real *V*_D_/*V*_T_ when anatomic or intrapulmonary shunts are present as it assumes a perfect *V*_A_/*Q* matching throughout all alveolar–capillary units [35, 36]. Although we used the VD_Enghoff_ method, we computed *V*_D_/*V*_T_ from the CO_2_ production ($V_{{{\text{CO}}_{2} }}$) and CO_2_ exhaled fraction ($F_{{{\text{ECO}}_{2} }}$) measurements by volumetric capnography, which can accurately reflect measurements by metabolic monitors [20].

Trying to exclude cases with transitory hypoxemia simulating ARDS, we completed a two-step selection previously described [16, 17]. Although breathing pure oxygen may influence the *V*_A_/*Q* distribution [5], our definitive *V*_D_/*V*_T_ measurements were performed 60 min after return to the previous FiO_2_, thus causing the lowest impact on *V*_A_/*Q* balance. Also, we used a stepwise recruitment maneuver with progressive PEEP increases, which was part of the local protocol at the time in which patients were included. Probably at present, such maneuver would not be used as recent evidence suggests that it can be harmful [37]. Nevertheless, such maneuver allowed us to standardize the selection of PEEP and ventilatory parameters.

We recognize that our study has important limitations. First, many hemodynamic and respiratory coexisting factors can influence *V*_D_/*V*_T_ measurements. Indeed, combination of hypovolemia, vasoactive agents and/or inotropics, cardiac output variations, pulmonary resistances and flows, distribution of ventilation along the lungs and even local microthrombi formation, might influence *V*_D_/*V*_T_ variations in one or other direction. Thereby, identical *V*_D_/*V*_T_ elevations might reflect simultaneous alterations in diverse physiological components. Second, our study is not able to demonstrate a causal association between *V*_D_/*V*_T_ and sublingual microcirculation and it was not registered as observational study. Nevertheless, dynamic variations from baseline to 24 h after merit exploration in future studies. Third, whether pulmonary microvascular alterations occur in parallel to other extra-pulmonary microvascular beds is highly controversial. However, microcirculation studies reveal simultaneous alterations at different beds during shock or inflammatory conditions. Fourth, the number of cases included in our study was relatively small. However, the fact that our patients were strictly selected and calculation of *V*_D_/*V*_T_ used $V_{{{\text{CO}}_{2} }}$ and exhaled fraction of CO_2_ ($F_{{{\text{ECO}}_{2} }}$) measurements by volumetric capnography strengthens our results.

## Conclusion

Increased heterogeneity of microcirculatory blood flow evaluated at sublingual mucosa seems to be related to increases in *V*_D_/*V*_T_ independently of respiratory mechanics and oxygen parameters, thus suggesting that microcirculatory alterations could be implicated in ventilation/perfusion mismatching during early ARDS.

The inverse dynamic relationships observed between sublingual microcirculation and dead-space ventilation poses a hypothetical pathophysiological mechanism during moderate and severe ARDS that deserves future research efforts.

## Supplementary information

**Additional file 1.** Microcirculatory assessement.

## Data Availability

The datasets generated and/or analyzed during the current study are not publicly available as recommended by the local Ethical and research committee involving human beings (Fundación Valle del Lili, Cali, Colombia) but these could be available from the corresponding author on reasonable request and under prior approval by such committee.

## Abbreviations

ARDS: Acute respiratory distress syndrome
CO_2_: Carbon dioxide
COPD: Chronic obstructive pulmonary disease
*C*_RS_: Compliance of the respiratory system
EtCO_2_: End-tidal CO_2_
FCD: Functional capillary density
$F_{{{\text{ECO}}_{2} }}$: Exhaled CO_2_ fraction
MFI: Microcirculatory flow index
$P_{{{\text{aCO}}_{2} }}$: Arterial CO_2_ partial pressure
$P_{{{\text{ECO}}_{2} }}$: Exhaled CO_2_ pressure
PEEP: Positive end-expiratory pressure
PPV: Percentage of small-vessels perfused
TCD: Total capillary density
*V*_A_/*Q*: Ventilation-to-perfusion ratio
$$V_{{{\text{CO}}_{2} }}$$: CO_2_ production
*V*_D_/*V*_T_: Dead-space ventilation fraction
*V*_E_: Exhaled minute ventilation
*V*_T_: Tidal volume
*V*_T_/*V*_RS_: Driving pressure
