# Supplementary material for: Microcirculatory dysfunction and dead-space ventilation in early ARDS: a hypothesis-generating observational study
Source: Ann Intensive Care. 2020 Mar 24;10:35. doi: 10.1186/s13613-020-00651-1 (PMC7093634; doi:10.1186/s13613-020-00651-1)
Supplement: Supplementary file 1 — Additional file 1. Microcirculatory assessement. [file 13613_2020_651_MOESM1_ESM.docx]

**MICROCIRCULATORY DYSFUNCTION AND DEAD-SPACE VENTILATION IN EARLY ARDS: A HYPOTHESIS-GENERATING OBSERVATIONAL STUDY**

**Gustavo A. Ospina-Tascón ^1^, Diego F. Bautista ^1^, Humberto J. Madriñán ^1^, Juan D. Valencia ^1^, William F. Bermúdez ^1^, Edgardo Quiñones ^1^, Luis Eduardo Calderón-Tapia ^1^, Glenn Hernandez ^2^, Alejandro Bruhn ^2^, Daniel De Backer ^3^.**

**Additional Material**

1. Department of Intensive Care Medicine, Fundación Valle del Lili - Universidad ICESI. Cali, Colombia
2. Traslational Medicine Laboratory for Critical Care and Advanced Trauma Surgery, Universidad Icesi. Cali,  Colombia
3. Departamento de Medicina Intensiva, Pontiﬁcia Universidad Católica de Chile. Santiago, Chile.
4. Intensive Care Department. CHIREC Hospitals, Université Libre de Bruxelles, Brussels, Belgium

**Address for correspondence:** Dr. Gustavo A. Ospina-Tascón

Department of Intensive Care Medicine

Traslational Medicine in Critical Care

and Advanced Trauma Surgery Laboratory

Fundación Valle del Lili - Universidad ICESI

Av. Simón Bolívar Cra. 98

Cali, Colombia

Tel (+57) 2 331 9090 –

Fax (+57) 2 331 9090 ext.4237

Email: [gusospin@gmail.com](mailto:gusospin@gmail.com)

**Microcirculation assessment**

A Sidestream dark-field (SDF) imaging device (Micro Scan; MicroVision Medical, Amsterdam, the Netherlands) was used to explore the sublingual microcirculation simultaneously to dead-space fraction measurements, ventilatory mechanics and oxygenation parameters at both inclusion and 24-hours after. This portable video-microscope device uses a stroboscopic green light (around 530 nm wavelength), which is delivered to the tissues by multiple light-emitting diodes (LEDs). This wavelength of light is absorbed by hemoglobin of red blood cells, allowing their observation as dark cells flowing in the microcirculatory net while the light reflected by superficial layers does not reach the optics. As result of peripheral location of LEDs and the synchronization between light emission and camera frame rate, SDF provides a detailed visualization of open capillaries using a 5x objective and providing an on-screen magnification of x380.

The SDF device was softly applied to the lateral side of the tongue covering an area approximately of 2 – 4 cm from the tip of tongue after gentle removal of secretions with gauze. Light intensity and focus were manually adjusted until obtain the best quality in each case. Operator of SDF device was a well-trained physician (H.J.M.) exclusively dedicated to collect data information in clinical and experimental trials concerning to microcirculatory alterations in our intensive care department. At each time of measurements, we collected five sequences of video of 20 seconds each from different adjacent mucosa areas using a videocard (MicroVideo; Pinnacle system, Mountain Views, CA, U.S.A.). These sequences of video were stored under a random number and later analyzed by two investigators blinded to the origin of sequences (H.J.M., G.O.T.). For analysis, a grill of three horizontal and three vertical lines was placed in front of the screen while each sequence of video was reproduced. The vascular density was calculated as the number of vessels crossing these lines divided by the total length of the lines. Total vascular density (TCD) corresponds to the total number of vessels (small and large, with and without normal flow) while functional capillary density (FCD) corresponds to the number of well-perfused small vessels (< 20 µm) per area unit. The type of flow was defined as continuous, intermittent, or absent. The vessels were separated into large (mostly venules) and small (mostly capillaries) vessels using a cut-off value of 20 μm in diameter. Vessel perfusion (total, large and small) was defined as the proportion of perfused vessels, calculated as the number of vessels continuously perfused during the 20-second observation period divided by the total number of vessels of the same type. Thus, we report the proportion of small vessels perfused (PPV) corresponding to the proportion of well-perfused vessels with a diameter < 20 µm (mainly capillaries). A heterogeneity index of microcirculatory blood flow was calculated as the difference between maximal and minimal PPV values in five different mucosa areas divided by its own mean value:

$$\frac{\left( {PPV}_{max}- {PPV}_{min} \right)}{{(PPV}_{1}+ {PPV}_{2}+{PPV}_{3}+ {PPV}_{4}+ {PPV}_{5})/5}$$

Additionally, a second grill consisting in one vertical and one horizontal line was placed in front of the screen to separate the image in four quadrants. Microvascular flow was characterized as absent (0), intermittent (1), sluggish (2), or normal (3). Average of values of those four quadrants was reported as microvascular flow index (MFI). The intra and inter-observer variability were calculated according to the reading of three sequences analyzed five times at twelve-week intervals by two observers (H.J.M. and G.O.T.). The coefficient of variability of the determination of one sequence ranged from 2.5 to 6.2% (intra-observer) and from 3.0 to 5.2 % (inter-observer) for the total number of vessels, and from 1.6 to 4.8% (intra-observer) and from 3.8 to 8.0% (inter-observer) for the proportion of perfused vessels (all sizes).

**Figure S1. Selection of patients**

**
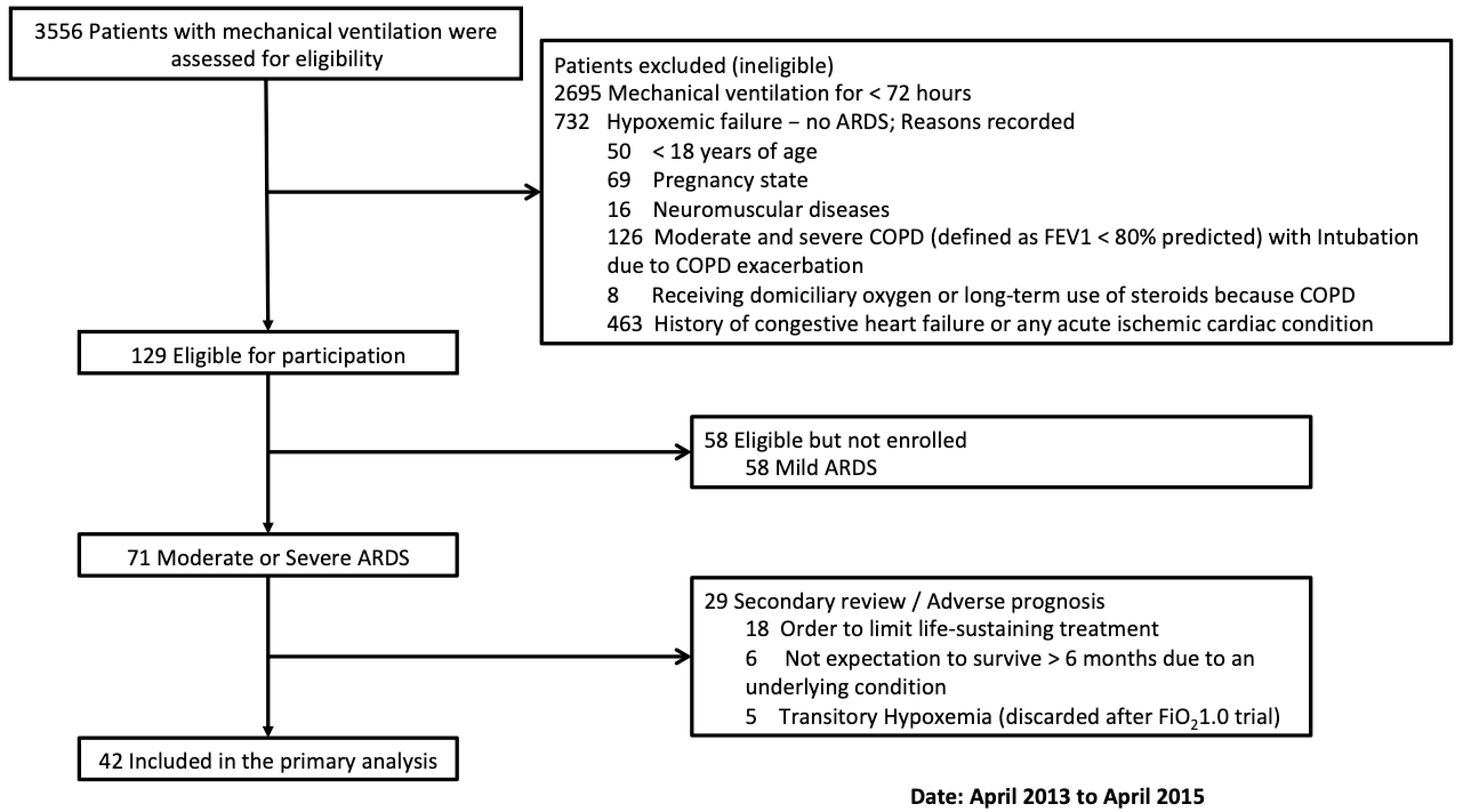
**

**Table S1.** ESTROBE Statement—Checklist for observational studies

|  | Item No | Recommendation | **Action / Fulfillment** |
| --- | --- | --- | --- |
| **Title and abstract** | 1 | (*a*) Indicate the study’s design with a commonly used term in the title or the abstract | It was specified in the abstract |
|  |  | (*b*) Provide in the abstract an informative and balanced summary of what was done and what was found |  |
| Introduction |  |  |  |
| Background/rationale | 2 | Explain the scientific background and rationale for the investigation being reported |  |
| Objectives | 3 | State specific objectives. including any pre-specified hypotheses |  |
| Methods |  |  |  |
| Study design | 4 | Present key elements of study design early in the paper |  |
| Setting | 5 | Describe the setting. locations. and relevant dates. including periods of recruitment. exposure. follow-up. and data collection |  |
| Participants | 6 | (*a*) Give the eligibility criteria. and the sources and methods of selection of participants. Describe methods of follow-up | Flow chart to select participants included in the ESM |
|  |  | (*b*) For matched studies. give matching criteria and number of exposed and unexposed | Do Not apply |
| Variables | 7 | Clearly define all outcomes. exposures. predictors. potential confounders. and effect modifiers. Give diagnostic criteria. if applicable |  |
| Data sources/ measurement | 8* | For each variable of interest. give sources of data and details of methods of assessment (measurement). Describe comparability of assessment methods if there is more than one group |  |
| Bias | 9 | Describe any efforts to address potential sources of bias |  |
| Study size | 10 | Explain how the study size was arrived at | Sample by convenience |
| Quantitative variables | 11 | Explain how quantitative variables were handled in the analyses. If applicable. describe which groupings were chosen and why | Partitioning by quantiles described in the text |
| Statistical methods | 12 | (*a*) Describe all statistical methods. including those used to control for confounding |  |
|  |  | (*b*) Describe any methods used to examine subgroups and interactions |  |
|  |  | (*c*) Explain how missing data were addressed |  |
|  |  | (*d*) If applicable. explain how loss to follow-up was addressed |  |
|  |  | (*e*) Describe any sensitivity analyses | Do Not apply |
| Results |  |  |  |
| Participants | 13* | (a) Report numbers of individuals at each stage of study—eg numbers potentially eligible. examined for eligibility. confirmed eligible. included in the study. completing follow-up. and analysed | Flow chart |
|  |  | (b) Give reasons for non-participation at each stage |  |
|  |  | (c) Consider use of a flow diagram | Provided in the ESM |
| Descriptive data | 14* | (a) Give characteristics of study participants (eg demographic. clinical. social) and information on exposures and potential confounders |  |
|  |  | (b) Indicate number of participants with missing data for each variable of interest |  |
|  |  | (c) Summarise follow-up time (eg. average and total amount) |  |
| Outcome data | 15* | Report numbers of outcome events or summary measures over time | Information about ICU. in-hospital and day-28 mortality is provided |
| Main results | 16 | (*a*) Give unadjusted estimates and. if applicable. confounder-adjusted estimates and their precision (eg. 95% confidence interval). Make clear which confounders were adjusted for and why they were included | Do Not Apply |
|  |  | (*b*) Report category boundaries when continuous variables were categorized | Information about categorization is provided |
|  |  | (*c*) If relevant. consider translating estimates of relative risk into absolute risk for a meaningful time period | Do Not Apply |
| Other analyses | 17 | Report other analyses done—eg analyses of subgroups and interactions. and sensitivity analyses | Sub-Analysis of patients using a Pulmonary artery catheter is provided |
| Discussion |  |  |  |
| Key results | 18 | Summarize key results with reference to study objectives |  |
| Limitations | 19 | Discuss limitations of the study. taking into account sources of potential bias or imprecision. Discuss both direction and magnitude of any potential bias | Discussed in main text |
| Interpretation | 20 | Give a cautious overall interpretation of results considering objectives. limitations. multiplicity of analyses. results from similar studies. and other relevant evidence | This is a generating-hypothesis study |
| Generalizability | 21 | Discuss the generalizability (external validity) of the study results |  |
| Other information |  |  |  |
| Funding | 22 | Give the source of funding and the role of the funders for the present study and. if applicable. for the original study on which the present article is based |  |

**Table S2. Bivariate correlations between some respiratory / microcirculatory variables and pulmonary dead-space fraction (V_D_/V_T_) and coefficients of determination (R^2^) for simple linear regression models predicting V_D_/V_T_ (with lineal and quadratic terms)**

|  | **Day 1** | | | | **Day 2** | | | |
| --- | --- | --- | --- | --- | --- | --- | --- | --- |
|  | **Spearman Rho** | **p value** | **R^2^ lineal** | **R^2^ quadratic** | **Spearman Rho** | **p value** | **R^2^ lineal** | **R^2^ quadratic** |
| **PPV** (%) | - 0.76 | < 0.001 | 0.60 | 0.63 | - 0.71 | < 0.001 | 0.66 | 0.66 |
| **MFI** | - 0.63 | < 0.001 | 0.48 | 0.48 | - 0.65 | < 0.001 | 0.54 | 0.60 |
| **HI** | 0.56 | < 0.001 | 0.36 | 0.43 | 0.55 | < 0.001 | 0.50 | 0.51 |
| **PaO_2_/FiO_2_ ratio** | - 0.19 | 0.22 | 0.02 | 0.09 | - 0.11 | 0.52 | 0.02 | 0.02 |
| **PEEP** | 0.15 | 0.33 | 0.04 | 0.04 | 0.09 | 0.58 | 0.01 | 0.03 |
| **Pm_aw_** | 0.05 | 0.78 | 0.02 | 0.02 | 0.10 | 0.53 | 0.01 | 0.01 |
| **V_T_/C_RS_** | 0.08 | 0.62 | 0.01 | 0.01 | 0.05 | 0.74 | 0.00 | 0.00 |
| **V_TE_** | - 0.11 | 0.48 | 0.01 | 0.01 | - 0.16 | 0.31 | 0.03 | 0.03 |

PPV: percentage of small vessels perfused; MFI: microcirculatory blood flow index; HI: heterogeneity Index; PaO_2_/FiO_2_ ratio: arterial oxygen partial pressure to oxygen inspiratory fraction; PEEP: positive end expiratory pressure; Pplat: plateau pressure of respiratory system, Pm_aw_: mean pressure of the airway; V_T_/C_RS_: tidal volume to respiratory system compliance ratio (i.e., driving pressure); V_TE_: expired tidal volume

**Figure S2. Relationships between dynamic variations in pulmonary dead-space fraction (V_D_/V_T_) and microcirculatory blood flow**


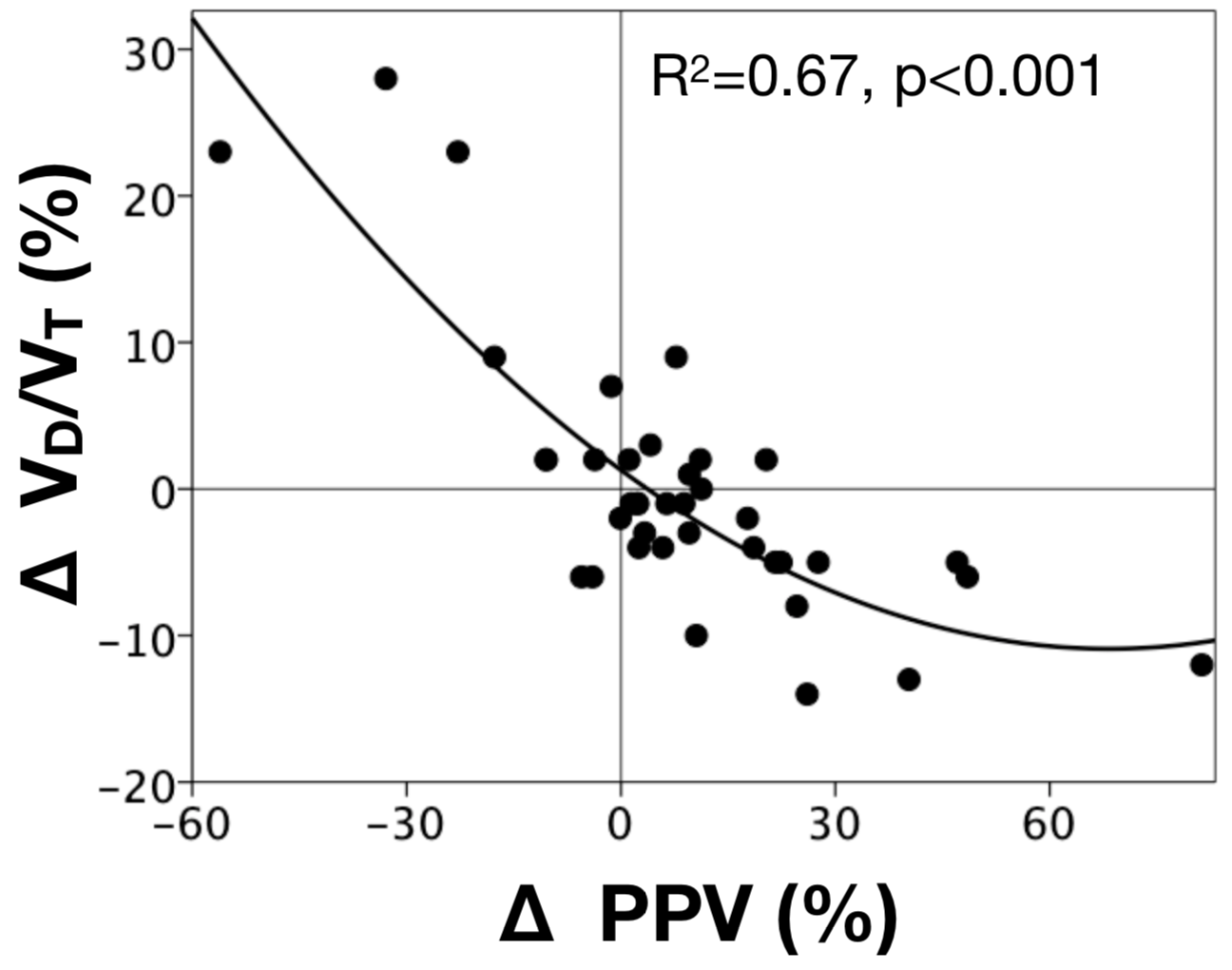


Scatter plot depicting the correlation between variations in pulmonary dead-space fraction (∆-V_D_/V_T_) vs. percentage of change in small vessels perfused (∆-PPV) between baseline and 24-hour after**.**

R^2^ represents the coefficient of determination for the linear regression with quadratic terms evaluating the correlation between the two terms.

**Table S3. Respiratory mechanics, blood gases analysis and pulmonary dead-space fraction for the entire population and for survivors and non-survivors at day-90**

|  | **All**  **N = 42** | **Survivors**  **N= 24** | | **Non Survivors**  **N= 18** | **P** | |
| --- | --- | --- | --- | --- | --- | --- |
| **Hemodynamics** | | | | | | |
| HR, bpm | 106 (94 – 125) | | 109 (97 – 125) | 104 (86 – 122) | | 0.49 |
| MAP, mmHg | 76 (70 – 87) | | 76 (70 – 87) | 72 (64 – 92) | | 0.43 |
| CVP, mmHg | 12 (8 – 14) | | 10 (8 – 13) | 13 (9 – 16) | | 0.27 |
| PAOP, mmHg | 14 (12 – 17) | | 14 (11 – 17) | 15 (14 – 16) | | 0.84 |
| PAPm, mmHg | 36 (32 – 46) | | 37 (34 – 46) | 36 (26 – 52) | | 0.82 |
| Cardiac index, (L/min/m^2^), n | 4.2 (3.4 – 4.5), 22 | | 4.2 (3.7 – 4.6) | 3.8 (3.1 – 4.5) | | 0.40 |
| Norepinephrine, (ugr/kg/min), n | 0.09 (0.05 – 0.36), 26 | | 0.06 (0.04 – 0.18) | 0.17 (0.05 – 0.80) | | 0.11 |
| **Respiratory mechanics and blood gases** | | | | | | |
| VT | 369 (324 – 416) | | 373 (315 – 420) | 363 (335 – 414) | | 0.59 |
| VT (ml/kg) | 6.5 (6.1 – 6.9) | | 6.5 (6.2 – 6.9) | 6.5 (6.1 – 6.9) | | 0.25 |
| RR | 24 (20 – 26) | | 24 (22 – 26) | 24 (20 – 26) | | 0.33 |
| PEEP | 13 (10 – 16) | | 12 (10 – 16) | 14 (10 – 15) | | 0.30 |
| Pm_aw_ | 19 (14 – 22) | | 20 (15 – 22) | 18 (14 – 21) | | 0.99 |
| VT/C_RS_ | 17 (14 – 19) | | 16 (14 – 18) | 17 (14 – 21) | | 0.65 |
| PaO_2_/FiO_2_ | 119 (93 – 152) | | 122 (99 – 156) | 115 (84 – 139) | | 0.07 |
| PaCO_2_ | 47.3 (38.9 – 56.7) | | 42.3 (35.8 – 49.9) | 52.1 (45.1 – 61.8) | | 0.05 |
| pH | 7.27 (7.20 – 7.31) | | 7.29 (7.22 – 7.34) | 7.23 (7.14 – 7.29) | | 0.002 |
| HCO_3_ | 20.8 (18.9 – 24.1) | | 20.6 (18.1 – 23.7) | 21.1 (18.9 – 24.4) | | 0.73 |
| BE | -6.6 (-9.2 – -2.4) | | -6.4 (-8.9 – -2.7) | -6.9 (-9.7 – -2.3) | | 0.27 |
| VD/VT | 41 (31 – 48) | | 35 (30 – 41) | 50 (44 – 56) | | < 0.001 |
| **Microcirculatory parameters** | | | | | | |
| PPV, % | 69.6 (56.6 – 79.1) | | 76.3 (70.5 – 83.6) | 57.8 (42.8 – 62.8) | | < 0.001 |
| LVP, % | 90.5 (80.0 – 96.8) | | 92.3 (84.2 – 98.0) | 87.3 (73.0 – 98.6) | | 0.15 |
| FCD, n vessels/mm^2^ | 6.6 (4.9 – 7.6) | | 7.4 (6.3 – 7.8) | 5.3 (3.4 – 6.6) | | 0.001 |
| TCD, n vessels/mm^2^ | 11.5 (10.7 – 12.6) | | 11.8 (10.8 – 12.6) | 11.3 (10.3 – 12.8) | | 0.62 |
| MFI | 2.4 (2.0 – 2.5) | | 2.4 (2.2 – 2.6) | 2.2 (1.7 – 2.5) | | 0.05 |
| Heterogeneity Index | 0.39 (0.27 – 0.68) | | 0.33 (0.25 – 0.63) | 0.51 (0.33 – 1.14) | | 0.03 |
| **Multiorgan dysfunction** | | | | | | |
| SOFA score, day-1 | 11.0 (7.8 -13.3) | | 8.0 (6.3 – 12.0) | 12.0 (11.0 – 14.0) | | 0.002 |
| SOFA score cardiovascular, day-1 | 3.0 (2.8 – 4.0) | | 3.0 (1.3 – 3.8) | 4.0 (3.0 – 4.0) | | 0.03 |
| SOFA score renal, day-1 | 1.0 (0.0 – 3.0) | | 1.0 (0.0 – 2.0) | 3.0 (0.8 – 4.0) | | 0.01 |
| SOFA score respiratory, day-1 | 3.0 (3.0 – 4.0) | | 3.0 (3.0 – 3.8) | 3.0 (3.0 – 4.0) | | 0.34 |

HR: heart rate; MAP: mean arterial pressure; CVP: central venous pressure; PAOP: pulmonary artery occlusion pressure; VT: tidal volume; PEEP: positive end-expiratory pressure; Pplat: plateau pressure of respiratory system; Pm_aw_: mean pressure of the airway; PaO_2_/FiO_2_ ratio: arterial oxygen partial pressure to oxygen inspiratory fraction; PaCO_2_: arterial CO_2_ partial pressure; Vd/Vt: pulmonary dead-space fraction; PPV: percentage of small vessels perfused; LVP: percentage of large vessels perfused; FCD: functional capillary density; TCD: total capillary density; MFI: microvascular blood flow index; SOFA: sequential organ failure assessment

**Sample size calculation**

Assuming a non-normal distribution, the sample calculation was based on the width of the Fisher confidence interval for the Spearman correlation ($\rho$) using the formula by Bonnet et al.*:

$$n=\left( n_{0}- b \right)\left( \frac{w_{0}}{w} \right)^{2}+ b$$

where,

$b$ is a constant equals 3 (in the case of Spearman correlation).

$n_{0}=4c^{2}\left( 1-\hat{\rho}^{2} \right)^{2}\left( \frac{z_{\alpha/2}}{w} \right)^{2}+b$, is the first approach to sample size.

$\hat{\rho}$ is a planning estimate of $\rho$, and $c^{2}=1+\frac{\hat{\rho}^{2}}{2}$

$\alpha$, represents type I error.

$w$, is the desired width of the Fisher confidence interval.

$w_{0}$, represents the width of the Fisher confidence interval obtained from $n_{0}$ as follows:

$$w_{0}=\left( \frac{{exp}^{2L_{2}}-1}{{exp}^{2L_{2}}+1} \right)-\left( \frac{{exp}^{2L_{1}}-1}{{exp}^{2L_{1}}+1} \right)$$

$$L_{1}=0.5\left[ Ln \left( 1+\hat{\rho} \right)-\ln\left( 1-\hat{\rho} \right) \right]-\frac{cz_{\alpha/2}}{\sqrt{(n-b)}}$$

$$L_{2}=0.5\left[ Ln \left( 1+\hat{\rho} \right)-\ln\left( 1-\hat{\rho} \right) \right]+\frac{cz_{\alpha/2}}{\sqrt{(n-b)}}$$

Thus, for a $\hat{\rho}=0.79$, a desired width $w=0.3$ and Fisher 95% confidence interval, we obtained a sample size of 37, which was finally extended to 42 assuming a loss of 10% of data.

**Reference**

* Bonett, D. G., & Wright, T. A. (2000). Sample size requirements for estimating Pearson, Kendall and Spearman correlations. *Psychometrika*, *65*(1), 23-28.
